# Geriatric patients' views on a pharmacist-led follow-up programme after discharge from hospital

**DOI:** 10.1016/j.rcsop.2025.100597

**Published:** 2025-03-25

**Authors:** Sofia Svahn, Gisselle Gallego, Maria Gustafsson, Marcia Håkansson Lindqvist

**Affiliations:** a Department of Medical and Translational Biology, Umeå University, Umeå, Sweden; b School of Medicine, The University of Notre Dame, Darlinghurst, NSW, Australia; c Department of Education, Mid Sweden University, Sundsvall, Sweden

**Keywords:** Geriatric patients, Transitions of care, Medication-related problems, Medication use, Follow-up programme, Clinical pharmacy

## Abstract

**Background:**

Medication-related problems (MRPs) are common during transitions of care and can lead to hospital readmissions. This patient safety issue is especially pronounced among geriatric patients. In a randomised controlled trial (RCT), the effect of a pharmacist-led follow-up programme after discharge from hospital for people ≥75 years in the north of Sweden was investigated. One of the components in the programme was telephone calls to study participants, to find and manage MRPs.

**Objective:**

To explore study participants' views on follow-up telephone calls by a clinical pharmacist in the RCT.

**Methods:**

Semi-structured interviews were conducted with participants who had received an intervention in the RCT. The interviews were transcribed verbatim and thematically analysed.

**Results:**

In total, nine participants were interviewed. Four main themes were generated: 1. Experiences of the telephone counselling by the clinical pharmacist, 2. Acceptability of receiving telephone follow-up from a clinical pharmacist, 3. Communication with health care providers, and 4. Medication management and views about medications.

**Conclusions:**

The study revealed varying perceptions of the clinical pharmacists' telephone calls, with participants expressing diverse experiences and preferences regarding the service. Most participants said they considered the content relevant and comprehensible in the conversations. The effect of the follow-up programme may have improved if the role of the clinical pharmacist had been explained in more detail to the participants and if the service would have had a more person-centred focus. More research is needed regarding how to best support geriatric patients with their medication treatment in transitions of care.

## 1 Introduction

The transition of care between different health care providers is a sensitive phase with an increased risk of medication-related problems (MRPs),^1^, ^2^, ^3^, ^4^, ^5^ such as non-adherence, adverse drug reactions and drug-drug interactions.^6^ Shortcomings in providing medication information at discharge can cause confusion and lead to MRPs when the patient returns home.^1^^,^^7^^,^^8^ Geriatric patients, who often have multiple medical conditions and complex medication regimens, are considered extra vulnerable in transitions of care.^4^^,^^5^^,^^9^^,^^10^ Hospital readmissions among geriatric patients are often medication-related and considered possible to prevent to some extent.^11^, ^12^, ^13^ It is a global challenge to minimise avoidable medication-related harm,^14^ and The World Health Organization has pointed out transitions of care as a central area where patient safety improvements can be made.^15^

The clinical pharmacist is one of the health care professionals who can contribute to improved patient outcomes such as quality of prescribing, reducing drug-related readmissions and all-cause readmissions.^16^, ^17^, ^18^, ^19^ In transitions of care, studies have found that pharmacists' interventions contribute to reducing medication discrepancies.^20^ Further, post-discharge interventions involving phone-based follow-ups by clinical pharmacists have reduced all-cause readmission rates.^21^^,^^22^ However, these studies did not specifically target a geriatric population. Evidence for an ideal intervention model in transitions of care is yet lacking, and more randomised controlled trials (RCTs) are needed and called for to investigate this field.^23^ In order to meet this research gap, an RCT is being conducted (clinicaltrials.gov: NCT03671629) in the north of Sweden addressing geriatric patients in transitions of care.^24^ The primary aim of the study was to investigate the effect of a clinical pharmacist follow-up programme in transitional care among people ≥75 years. The programme included several different actions, one of which was follow-up telephone calls with participants, by the clinical pharmacist to identify and manage MRPs.

The participant is central in the described intervention study, and patient satisfaction can affect patient outcomes. Patient experience has been positively associated with both self-rated and objectively measured outcomes, such as adherence to medication treatment, adverse events and hospitalisation.^25^ A better understanding of the participants' perspectives can give deeper insight into what influences the effects of the clinical pharmacist intervention. Previous qualitative studies have described participants' views of pharmacist-led follow-up programmes.^26^, ^27^, ^28^ A Swedish study aimed to investigate patients' experiences of an RCT including both medication reviews on medical wards and follow-up telephone calls by clinical pharmacists after discharge among people 65 years and older. The study found that patients generally had a positive view on these interventions.^26^ Similar results were found in a Norwegian study that explored patients' experiences of a pharmacist-led follow-up program including consultations after discharge, among people with coronary heart disease.^27^ In a study from the Netherlands, the aim was to identify barriers and facilitators with medication use among patients included in a pharmacist-led study during the transition from hospital to home. Participants expressed a lack of personalised care and insufficient information transfer between health care providers. Follow-up care after hospital discharge was seen as an important facilitator.^28^ As far as known, few studies have focused on geriatric patients' experiences of follow-up programmes run by clinical pharmacists in transitions of care, especially very old people. Therefore, the aim of this study was to explore the geriatric patients' views regarding follow-up telephone calls by a clinical pharmacist after hospital discharge.

## 2 Ethics

This study complies with the Declaration of Helsinki, and an ethics approval was obtained from the Swedish Ethical Review Authority (Reg.no. 2020–03771). The participants were provided with information about the study before they gave their verbal consent for participation and publication of their responses. Data were de-identified and participants were given a pseudonym to protect their identity.

## 3 Method

### 3.1 Study design

This descriptive qualitative study used semi-structured interviews conducted with a combination of an inductive and deductive data analysis. An interview guide was used to explore the opinions, feelings and perspectives of the participants. The Consolidated Criteria for Reporting Qualitative Studies (COREQ) guidelines^29^ were followed to report the study.

### 3.2 The RCT

The participants in this study were recruited from an ongoing RCT targeting people ≥75 years old, admitted to the medical ward at Umeå university hospital in the north of Sweden.^24^

The RCT investigated if a clinical pharmacist follow-up programme during transition of care could reduce medication-related hospital readmissions within 30 days and 180 days, respectively, after discharge compared to conventional care. The study commenced in September 2018 and will continue until 700 participants are included. The inclusion criteria were people ≥75 years old, living at home, emergency admission and being registered at one of nine specified primary care centres. Exclusion criteria were lack of knowledge in Swedish or difficulties in communicating, admission due to intoxication (drug or alcohol) or being scheduled for palliative care. A clinical pharmacist visited the individuals who met the inclusion criteria in the involved ward. The potential participants received information about the study and were invited to take part. Written informed consent was obtained from all included participants. Once the participant was discharged from the hospital, the follow-up time for that person began. No information regarding medications was provided by clinical pharmacists to participants in the ward.

Activities performed by the clinical pharmacist during the 180-day follow-up time included three key components. These were 1) regular reviews of medical records 2) medication reviews, if considered necessary, and 3) telephone calls to study participants. More information regarding medication reviews is provided in the study protocol of the RCT.^24^ During inclusion, participants were informed about the different components in the RCT. The participants received three scheduled telephone calls in total. If necessary, extra telephone calls were made to inform the participant about a change in medication use, as a consequence of an intervention or medication review performed by the clinical pharmacist. The first scheduled telephone call occurred within 7 days after hospital discharge. A structured guide was used by the clinical pharmacists during telephone conversations with patients. In these conversations, any changes made during or after hospitalisation in the medication treatment were discussed, medication reconciliation was carried out and individual counselling was provided regarding for example medication management, adherence and potential MRPs such as adverse drug reactions and drug interactions. If MRPs were identified, the clinical pharmacist would try to resolve these with the participant or in collaboration with prescribers when necessary. The pharmacist documented data from the conversations and any issues or problems patients were experiencing with their medications/illnesses as well as interventions on a record form. This included issues regarding practical handling (such as packaging, use of facilities, potential swallowing problems), medication adherence, potential adverse events, potential discrepancies including over the counter products. Examples of interventions by the clinical pharmacist included but were not limited to: clarifying information or dosing instructions that a participant had misunderstood, correcting discrepancies in medication lists (compared to what should really be taken by the participant), altering dose or choice of medication due to interactions or impaired renal function, and replacing a medication that the participant experienced adverse events from.

The clinical pharmacists involved in the RCT either had a degree from a one-year post-graduate programme in clinical pharmacy or completed undergraduate courses in clinical pharmacy and advanced pharmacotherapy. The clinical pharmacists did not receive any specific communication skills training before the RCT began.

### 3.3 Participant sampling and recruitment

Purposive sampling was used to identify participants who had received a clinical pharmacist intervention in the RCT. Interventions made specifically for the interviewed participants were, for example, switching to an angiotensin receptor blocker (ARB) from an angiotensin-converting enzyme (ACE) inhibitor due to side effects, and switching from one selective serotonin reuptake inhibitor (SSRI) medication to another to avoid an interaction. Clarification of misunderstood information was a frequent intervention, for instance, to a person who had taken double dose of a medication, and two persons who had not perceived that medications were discontinued at the hospital. People with cognitive impairment or other conditions (such as speaking difficulties after stroke) which made conducting the interview difficult were excluded.

The eligible participants were sent a letter of invitation (*N* = 16). About one week later, the interviewer [MHL] made a telephone call to formally ask if potential participants were interested in participating in the study. Participants provided verbal informed consent before the start of the interview. The recruitment and interviews were conducted between December 2021 and September 2023. The interviewer [MHL] was a new acquaintance to the participants, and had not been involved in the RCT.

### 3.4 Data collection

In-depth semi-structured telephone interviews were conducted with participants in the present study 3–6 months after inclusion in the RCT. The time interval was chosen to avoid too much interference with the RCT, in which the actions within the follow-up programme was most intense during the first three months. This type of interview was selected as it gives participants the opportunity to expand their answers freely and provide in-depth reflections about their experiences.^30^ An interview guide was developed and focused on four topics: *Experiences of the conversations with a clinical pharmacist, Experiences of the counselling with by the clinical pharmacist, The transition from hospital to primary care and how information regarding medications in those environments is perceived,* and *Acceptability of receiving counselling* via *telephone*. The interview guide was pilot-tested with one participant (not included in the analysis), and minor adjustments, such as question order, were made before the first interview. The participants were encouraged to speak freely, and they were given the chance to express their own thoughts and reflections before the interview ended.

In total, nine interviews were conducted by one researcher [MHL], and the interviews lasted up to 25 min. The interview process continued until data saturation occurred, when no new ideas or themes were generated.^31^ The interviews were digitally recorded, transcribed verbatim, checked for accuracy by one researcher [SS], and subsequently analysed [MHL and SS]. Each participant was assigned a unique participant code to de-identify the interview data. The interviews were conducted and analysed in Swedish and then translated into English. Quotations from the interviews are presented to illustrate the interpretation of themes. The participants are identified as Participant 101–116 (P101-P116).

The data were analysed using Braun and Clarke's six phases of thematic analysis.^32^^,^^33^ The analysis was a combination of an inductive and deductive analysis. In order to capture the perspectives of the participants, the topics in the interview guide were used as a starting point. To ensure coding reliability, the initial coding was done independently by two researchers [MHL and SS]. The process involved several discussions to reach consensus. Further analysis into themes and sub-themes was drafted by SS. Meetings were held frequently between SS and MHL to review and discuss the analysis to agree on thematic development. Excel, Microsoft 365, was used for data management.

SS (MSc) is a pharmacist with many years' work experience. SS is one of the clinical pharmacists involved in the RCT, and received training in qualitative analysis prior to the study. MHL (PhD) has a background in educational sciences, and has extensive experience of qualitative methods.

## 4 Results

Of the total 16 persons who were invited, nine participants gave their consent and were interviewed. Three people did not answer, two declined to participate and two did not remember the interaction with the clinical pharmacist. Average age of participants was 81.3 years (range 75–89) and all except one were females. Participant characteristics are presented in Table 1. All participants received three scheduled telephone calls during the RCT. Four different clinical pharmacists had been involved in the telephone counselling with participants.

**Table 1:** Participant characteristics (*N* = 9) at discharge from hospital.

Code	Age	Sex	Number of medications	Cardiovascular disease⁎	Diabetes mellitus	COPD	Cancer
106	75	Female	3		x		
108	82	Female	6	x	x		
109	80	Female	11	x	x		
110	78	Female	7	x			
111	79	Female	11	x	x		
112	89	Female	8	x		x	x
113	78	Female	9	x			
115	86	Male	6	x		x	
116	85	Female	9	x			x

COPD = Chronic obstructive pulmonary disease.

⁎ Cardiovascular disease include: Atrial fibrillation, atherosclerotic cardiovascular disease, heart failure and hypertension.

In the analysis of the interviews, four key themes were identified: *1. Experiences of the telephone counselling by the clinical pharmacist, 2. Acceptability of receiving counselling* via *telephone, 3. Communication with health care providers* and *4. Medication management and views about medications*. Identified themes and subthemes are presented and exemplified with quotes.

### 4.1 Theme 1. Experiences of the telephone counselling by the clinical pharmacist

The participants were asked about their experiences of the counselling provided by the clinical pharmacist in the telephone calls. Based on their responses, two subthemes were generated: *Content and perceived value from the counselling* and *Competence, communication skills and the role of the clinical pharmacist*.

#### 4.1.1 Subtheme. Content and perceived value from the counselling

All participants had received counselling and some kind of intervention from the clinical pharmacist resulting in changes in treatment or clarification of information regarding their medications. Nevertheless, many of the participants were not aware of the clinical pharmacist's actions, or did not remember:

“I don't remember, to be honest. […] But no, I don't think we discussed anything in particular because it's not necessary.” (P116).

A few participants could describe what was mentioned in the counselling and the outcome. These persons reported that their treatment had improved. One participant mentioned that she was able to discuss her treatment with the doctor after the clinical pharmacist contacted the prescriber: “I got direct contact with a doctor who simply removed the medication that was not suitable at the time, and that was good” (P113). Another participant talked about his medication changes, initiated by the clinical pharmacist:

“I think there was some change in something, some medicine that I complained about. Among other things, a medicine that I couldn't sleep on, but then I could switch in the end, and now I sleep well.” (P115).

The same participants provided examples of what they perceived as helpful advice or information regarding their medications during the counselling by a clinical pharmacist: “It's a push in the right direction [...] I've had help with this a couple of times and it gives you confidence” (P113) and: “It was good, because then they could describe the medicine, because you're pretty bad at reading the description yourself” (P115).

Participants' views regarding the value of counselling by a pharmacist varied. In contrast to the participants who remembered and appreciated the help they had received, some participants did not think that they gained anything at all, and some were unsure regarding potential benefits: “In practical terms, I have not had that much use of it” (P109). Others mentioned they could potentially benefit from the counselling, and that they appreciated the possibility to ask questions: “I hope that it is of some use that you have had these conversations and then you have a chance to ... well, ask if there is something [you need to ask]” (P112).

The participants expressed diverse views about the knowledge they gained about their medications through counselling. Some participants thought they had acquired information that was new to them via the clinical pharmacist**:** “Yes, I suppose it was [new knowledge]” (P108), as well as there were participants who said they did not gain any new knowledge about their medications: “No, I can't say that I have gained [new knowledge], no more than what I already have, so to speak” (P116).

#### 4.1.2 Subtheme: Competence, communication skills and the role of the clinical pharmacist

All participants said they trusted the clinical pharmacist's competence in providing counselling. The pharmacists were described as knowledgeable and supportive in their counselling. The content was considered relevant, comprehensible and reliable by most participants.

The overall view of the clinical pharmacists' communication skills was good. All participants described the communication as effective, and that it was easy to understand each other. The clinical pharmacist's ability to use plain language, as well as being able to listen and explain was highlighted by one participant: “They talk so you understand, even though you're old [...] If there's something you don't quite understand, just ask, they know [...] I thought they listened well and explained.” (P112).

Despite the above-mentioned positive views of the clinical pharmacists' competence and approach, the role of the clinical pharmacist seemed to be unclear to several participants. A general expectation was that receiving, and/or asking for counselling regarding medications takes place at the community pharmacy. This was spontaneously mentioned by several participants even though there were no questions about community pharmacies in the interview guide: “At present, you get very good advice at the pharmacy.” (P106) and “I am satisfied with the pharmacy I go to.” (P111).

### 4.2 Theme 2. Acceptability of receiving telephone follow-up from a clinical pharmacist

The majority of participants thought that the telephone follow-up was good, and the extent of the conversations was satisfactory. All participants said they had a chance to ask the clinical pharmacist questions. One participant mentioned that she appreciated having enough time to think about if there was something she was wondering about regarding her medications, and get answers to her questions from the clinical pharmacist: ”Great, because of course you can have a lot of questions […] about medication and if they change medication and stuff like that. […] You had time to think through if you had any questions and so on” (P109). Conversely, one of the participants said that there was no need for the service of telephone counselling by the clinical pharmacist: “No, I do not know if it is necessary. No, I don't think so. Not as long as you have legs and can walk there yourself. To the pharmacy.” (P111). Most of the participants expressed that there was “just the right amount of phone calls” (P108). However, there were two participants who thought the phone calls came too often: “Yes, I thought it was a bit often, because she probably called once a month for, I don't know, four months maybe [...] my answers were the same every time, so she could have reduced those calls to one.” (P110).

Most of the participants thought that follow-up via telephone calls worked well. Some of them thought the follow-up was best conducted via telephone:”No, but the telephone is perfect in this day and age, you shouldn't have personal meetings.” (P110) The interviews took place during the COVID pandemic when people were advised to avoid in-person meetings. A few participants mentioned that face-to-face would be good, one of which would have appreciated a home visit: “It's always easier to meet a person and see a person and talk to them, than on the phone, I think, but I have nothing against either.” (P113). Video meetings were suggested as an alternative by one participant, and another participant expressed that he would have preferred an in-person meeting over telephone calls: “Yes, of course, a personal meeting is best but takes a lot of time for everyone” (P115).

### 4.3 Theme 3. Communication with health care providers

This theme included two subthemes: *Information about medications in the medical ward* and *Difficulties in communicating with health care providers.*

#### 4.3.1 Subtheme. Information about medications in the medical ward

A few of the participants could not recall whether they received information about their medications during their stay in the medical ward, and some participants said they did not receive information, “not a bit” (P110), about their medications. One of them seemed disappointed about the lack of information: “When you get to the hospital, they take over the medication and then you just get a little plastic cup and there it all is. You just have to swallow it. I got no information. That was bad.” (P115). Another participant who did not get information regarding her medications said she “trusted the hospital staff to give her the right medications” (P116). This participant wished the hospital staff had given her some more information: “Yes, the *why* ... for the different medications, what their effects are a little bit.” (P116).

Some participants thought they received just the right amount of information about their medications in the ward, and several participants were happy with not getting any information about their medications while they were admitted. One of the reasons mentioned was that there were no changes made on the medication list: “No [when asked if she wanted to get information in the ward], because: ‘Yes, you can go home and it's the same medication as before’.” (106). A few participants did not want to receive information about medications in the ward, because they thought they were not in a state to take in information at that time: “When you're admitted, you don't feel so good. I don't know if you are that receptive then.” (P111).

#### 4.3.2 Subtheme. Difficulties in communicating with health care providers

A few participants reflected on their experience of problems in communicating with health care providers. Especially, the electronic communication system, referred to as “1177”, was emphasized in this context. The name 1177 is used for the Swedish health care system's website and telephone helpline. Via the personal pages on the 1177 website, it is possible for individuals and health care providers to interact regarding appointments, electronic health records and more. Reaching primary care centres was considered difficult by one participant:

“I don't know ... maybe you're supposed to go to 1177 and read everything there now. It's complicated sometimes to get in and find your way around.” (P115).

Another participant commented that referrals or booked appointments had gone missing in the system.

### 4.4 Theme 4. Medication management and views on medication

All the comments and views on medication management were expressed by participants without being asked by the interviewer.

Generic substitution was brought up as problematic. One participant described frustration related to the constantly changing names of the medications, and worries that it might be a problem keeping the medications apart in the future:

“I think I was talking to this pharmacist about that: ‘I'm going crazy, you never get ... the medicine is never called the same twice.’ This, I think it's horrible, and: ‘Where am I going to find the information that this is the one that replaces?’, and my mind is still pretty fresh, but: ‘How is this going to be?’ [...] Why should we have these constant changes? You never get the same one twice, but you change to a different one each time.” (P106).

Another participant talked about having problems with certain packages of medication, and wished all medications would be available in bottles rather than blister packs:

“…it's not you, but the fact that you can put it forward is that you stop having ... that you get medicines in bottles instead of these damn aluminium packages. And just imagine, yes, my fingers are a bit sore. I've sat and pushed them out for me, and then I have to distribute my medicine to my partner, and then I can imagine that those who are even worse in their hands, how do they do? [...] I find it difficult, so much easier to open a bottle and then you get it.” (P109).

Some of the participants freely started talking about their good general overview of medications: “I have a pretty good idea of what medications I take.” (P109) and that they are careful with their medications:

”First, I am careful with my medication … I hope I am prepared for the day if I become forgetful, that I get help and there is help available, to sort and so on … just one of those things that you actually can have it sent home packed and ready” (P113).

A common opinion among the participants was that they had too many medications and/or did not want to take so many medications: “I have an awful lot of medicines. I take a lot of medicines.” (P111). Another participant expressed the idea of under-dimensioning medications: “It's more likely that I might be under-sizing, or want to be, well, not take so much.” (P110).

A few participants brought up their curiosity about their medications and being updated, and said they ask questions if necessary: “...if you're curious and ask, you can also ask at the regular pharmacy, they always tell you if there's any difference between the manufacturers and everything that's going on, you listen to it.” (P112). Another participant asked the interviewer questions: “...for what I would like to ask. That is what they are for.” (P116).

## 5 Discussion

This study explored geriatric patients' views regarding follow-up telephone calls by a clinical pharmacist after hospital discharge. Many participants appreciated follow-up telephone calls by a clinical pharmacist. This aligns with positive patient experiences in previous studies of pharmacist involvement in post-discharge follow-up programmes.^26^, ^27^, ^28^ The extent and content of conversations was perceived as adequate by most participants in the present study. The possibility to ask questions was repeatedly mentioned as a positive aspect in the participants' experiences of the telephone calls, as well as being provided information regarding their medications. Most participants thought the communication worked well, and that the counselling was valuable. However, there were participants who thought they already knew enough about their medications, or that the service was not needed. This variation, in addition to the diverse opinions regarding suitable amount of information received at the hospital ward, suggest that information about medications should have been more personalised. In the RCT, standardized forms were used for the follow-up telephone calls. Some of the content may have seemed repetitive to persons who were not having any difficulties in medication management or were not experiencing any MRPs. To individualise the content, maybe some questions in the forms could have been reduced for participants with few medications and/or medical conditions. Participants could also have been asked about how much they knew about their medications and how interested they were in receiving help. The importance of tailoring information to suit each individual's needs and preferences has been described in previous studies.^8^^,^^28^^,^^34^ Person-centered pharmaceutical care has shown positive results in some randomised controlled trials.^35^, ^36^, ^37^

According to patients in the study by Daliri et al. (2019), facilitators for better medication use during transitions of care would be having personal medication counsellors as well as post-discharge follow-up in the form of telephone calls or home visits.^28^ Further, they suggested that the counsellor could serve as a point of contact between the patient and health care providers. This is very similar to the service provided within the RCT, as the counselling was adapted to each participant and their medications. The majority of participants thought telephone was the best way to communicate, however, one participant would have preferred a home visit instead. Clinical pharmacists in the follow-up programme could be seen as a link to health care providers, since there were reports of difficulties reaching and communicating with health care providers. Some participants described that the clinical pharmacist made it easier to get in touch with the doctor, or that the clinical pharmacist managed contact with the prescriber to resolve MRPs. Nevertheless, providing person-centered care can be difficult for health care providers.^38^ In the provided follow-up programme, content in the counselling was probably not successfully tailored to suit everyone. Some participants thought the telephone calls were not necessary or could have been reduced to fewer calls.

Although all of the participants had received some sort of intervention and/or clarification of information regarding their treatment, only two of them were aware of the clinical pharmacist's actions regarding the management of their medications. Since most participants did not remember what the pharmacist had accomplished regarding improvements in their treatment, it is difficult to evaluate the effect of the pharmacist's intervention. Kempen et al. (2020) showed similar results, where the patients could not differentiate if the changes were made as a result of the clinical pharmacist's involvement or usual care.^26^

It could be speculated that unawareness of the intervention is connected to participants' limited understanding of the clinical pharmacist's role. This probably reflects the fact that clinical pharmacists are not a common feature in health care in this part of Sweden, and most people have not encountered a clinical pharmacist before. Several participants expressed expectations that counselling takes places in a community pharmacy, which could indicate that most people expect pharmacists to work at a community pharmacy and not in other health care settings. If the participants did not know that the clinical pharmacist, for example, had access to medical records and was able to collaborate with prescribers to make changes in drug therapies, it is understandable that participants did not notice what the clinical pharmacist had accomplished. The unclear role of the pharmacist might also have had a negative effect on the outcomes of the follow-up programme as the participants did not know what the pharmacist could help them with. The unclear role of the clinical pharmacist among patients has been observed in previous studies.^26^^,^^27^^,^^39^^,^^40^ It has also been considered a risk for limiting the potential of getting the most benefit from health care systems.^39^ The effect of the intervention may have improved by explaining the pharmacist's role to the participants more thoroughly at the time of inclusion. To make it easier for participants to know what to expect and ask from the clinical pharmacist, examples of possible interventions could have been provided. It could also have been helpful to inform them that the clinical pharmacist had access to complete medical records in contrast to community pharmacists who only have the list of prescribed medications. Meanwhile, it is difficult for patients to differentiate between different health care professionals.^41^ A recent study suggested it may not be important to the patient which professional actually performed the service, as long as the patient felt taken care of.^40^

Generic substitution was highlighted by some participants as problematic and causing distress. This has been reported in previous studies,^26^^,^^28^^,^^42^, ^43^, ^44^ and has been suggested to increase the risk of medication errors among geriatric patients.^43^^,^^44^ There were different electronic systems for medical records at the hospital and dispensing of medications at community pharmacies. Information regarding dispensed medications was therefore not available for the clinical pharmacist, which limited the ability to help the participants with this issue.

### 5.1 Strengths and limitations

The main strength of this study was the focus on geriatric patients' perspectives on follow-up telephone calls post-discharge. Geriatric patients are often excluded in research relevant to their health and care.^45^ This qualitative study provided in-depth, rich data exploring geriatric patients' experiences, and provided ideas regarding how to improve similar services, which may lead to better outcomes.

There are some limitations that should be acknowledged. The small sample size is a limitation. However, saturation was reached during the conducted interviews. Also, as mentioned above, qualitative studies rarely include geriatric patients, especially ≥75 years. Thus, these findings still make a valuable contribution. As recruitment of the participants took longer than expected, and to complete the interviews within reasonable time, the sample ended up consisting of almost exclusively women. All participants had been admitted to the medical ward at one hospital. Participants may have been healthier than the average participant in the intervention study, since persons with cognitive decline and difficulties communicating were excluded. Also, the RCT patients may have experienced cognitive overload, when receiving information in hospital. The above-mentioned factors may have influenced the results, as well as the transferability of the findings.

Respondents in this interview study were contacted for participation 3–6 months after inclusion in the RCT, to avoid interference with the RCT. This, however, could have contributed to participants poor recall of the clinical pharmacists' interventions.

One of the researchers [SS] performing the analysis was involved in the RCT, which may have influenced the interpretation. To address this, quotations were included to validate the generated themes. It is also possible the researchers' preconceptions may have influenced the study. However, the use of reflexivity through reading and re-reading in reflection was used to create distance regarding the decisions and actions taken throughout the duration of the study.

## 6 Conclusion

The study revealed varying perceptions of clinical pharmacists' telephone calls, with participants expressing diverse experiences and preferences regarding the service. Most participants said they trusted the clinical pharmacist's competence and considered the content relevant and comprehensible in the conversations. The clinical pharmacist could be seen as a link to health care providers. By tailoring the provided service to better suit each patient's needs and preferences, the effect of the follow-up programme may have improved. Giving the participants more information about the clinical pharmacist's role would probably also have had a positive effect on the outcomes.

This study highlights the importance of resolving and explaining MRPs in a way that patients understand and can benefit from. When developing future services, ensuring a patient-centered approach should be a priority. Further studies could also include a comparison of the effectiveness of follow-up methods, such as telephone calls, virtual meetings and physical meetings.

## CRediT authorship contribution statement

**Sofia Svahn:** Writing – review & editing, Writing – original draft, Project administration, Methodology, Formal analysis, Conceptualization. **Gisselle Gallego:** Writing – review & editing, Methodology. **Maria Gustafsson:** Writing – review & editing, Supervision, Project administration, Methodology, Funding acquisition, Conceptualization. **Marcia Håkansson Lindqvist:** Writing – review & editing, Supervision, Methodology, Formal analysis, Conceptualization.

## Funding

This study was supported by grants from Region Västerbotten (Sweden), the 10.13039/501100007687Swedish Society of Medicine, 10.13039/501100004885Umeå University Foundation for Medical Research, Swedish Research Council for Health, Working Life and Welfare (Forte) (ref: 2017–01438) and the 10.13039/501100004359Swedish Research Council (ref: 2019–01078).

## Declaration of competing interest

The authors declare that they have no known competing financial interests or personal relationships that could have appeared to influence the work reported in this paper.

## Data Availability

The datasets generated and/or analysed during the current study are not publicly available due to issues of individual privacy but are available from the corresponding author on reasonable request.
